# Can We Blame Migratory Birds for Transmission of the Emerging Severe Fever with Thrombocytopenia Syndrome Virus in East Asia?

**DOI:** 10.4269/ajtmh.15-0524a

**Published:** 2015-12-09

**Authors:** Chang-Yong Choi, Chang-Wan Kang, Young-Min Yun, Hyun-Young Nam

**Affiliations:** Center for Spatial Analysis University of Oklahoma Norman, OK E-mail: subbuteo@hanmail.net; Jeju Wildlife Research Center Hawon-dong, Seogwipo Jeju, Republic of Korea E-mail: jejubirds@hanmail.net; College of Veterinary Medicine Jeju National University Jeju, Republic of Korea E-mail: dvmyun@jejunu.ac.kr; School of Biological Sciences Seoul National University Seoul, Republic of Korea E-mail: stern0223@lycos.co.kr

Dear Sir:

We read with interest the article by Yun and others entitled “Phylogenetic Analysis of Severe Fever with Thrombocytopenia Syndrome Virus in South Korea and Migratory Bird Routes between China, South Korea, and Japan” recently published.^1^ The authors proved that the severe fever with thrombocytopenia syndrome virus (SFTSV) isolated in South Korea shares a clear phylogenetic link with samples from Japan and China,^1^ indicating linkage of the viruses across outbreak areas in three east Asian countries separated by sea, an ecological barrier for terrestrial host and vector animals. To delineate transmission across the countries, Yun and others suggested that migratory birds play an important role in the new introduction of SFTSV in east Asia.^1^ We understand that they reached the conclusion based on four reasons: 1) migratory birds (especially four avian species) may carry ticks and associated pathogens, including *Haemaphysalis longicornis* that is known as a vector of SFTSV and has been collected from birds in Korea, 2) high-prevalence regions of SFTSV concur with geographic ranges of migratory birds in east Asia, 3) patients had no history of international travel, and 4) terrestrial mammals cannot cross the sea.

Migratory birds can obviously carry parasitic arthropods and associated pathogens,^2^,^3^ and we agree with the authors' opinion that migratory birds are one of the possible pathways of SFTSV introduction.^1^ However, there are some key remaining questions considering migratory birds as vectors of SFTSV.

First, SFTSV has a very confined distribution in the context of the spatial scale of bird migration. Migrations of birds are generally “latitudinal” movements in a predictable manner in terms of spatial and temporal scales.^4^,^5^ The regions where SFTS patients were reported lie at similar latitudes (between 30° N and 40° N) in a temperate zone, whereas many migratory birds (including the four species reported by Yun and others^1^) regularly exceed this range for breeding and wintering.^5^ If the transmission is mainly caused by bird migration, SFTS patients should be more widely (latitudinally, not longitudinally) distributed along the east Asian flyway of birds (i.e., southern China, southeast Asian countries, and the Russian Far East). In particular, migratory birds often have smaller and confined nonbreeding ranges in warm climate zones, with an increased population density during wintering periods.^5^ Nonbreeding areas of migratory birds at lower latitudes are potential habitats for ticks to find other host and to survive in winter. Therefore, given the lack of patient reports from northern and especially southern tips of migratory bird ranges, we believe that SFTSV can be regarded as a localized disease in terms of avian migration. In addition, we do not fully understand how the ecology (migration pattern, timing, routes, and connectivity) of migratory birds in east Asia, especially of migratory songbirds,^4^,^5^ explains the transmission of SFTSV.

Second, there are clear differences between ticks infesting mammals and birds in terms of species and their life cycles. Positive SFTSVs were mainly detected in adult female ticks, especially, *H. longicornis*.^6^ However, adult stages of any tick species are rarely found on birds.^3^ Unlike *Haemaphysalis flava* that was a dominant tick collected from migratory birds^3^ and was free from SFTSV,^6^ *H. longicornis* was an accidental tick on birds even in *H. longicornis*-dominated landscapes, suggesting that ticks on birds can be very different from those on mammals.^3^ Indeed, SFTSVs have been mostly detected in ticks from mammals and vegetations.^6^

Finally, the occurrence of SFTS in patients without international travel history is consistent with infection and transmission largely caused by local mammals. Although terrestrial wild and domestic mammals carrying SFTSV cannot cross the sea in a natural condition, we cannot ignore anthropogenic factors in animal movements: increasing international trade of agricultural products and domestic and exotic animals. We may pretend that customs and quarantine services completely regulate introduction of ticks, but there are still large volumes of undetected movements of animals through illegal wildlife smuggling from China to Korea and Japan.^7^ Even though illegal trades are mostly focused on wildlife products such as ivory and rhino horn, increasing demand for exotic pet animals through internet-based trade enables long-distance movements of high-value wildlife and live animal smuggling in east Asia.^7^

We respect the conclusion of Yun and others^1^ that migratory birds are possible contributors to the clear phylogenetic link for SFTSV across Korea, China, and Japan. We also support the authors' suggestion that detection and isolation of SFTSV in bird ticks and serological surveillance for SFTSV in migratory birds are required.^1^ Nevertheless, we currently have little evidence of long-distance transmission of SFTSV through bird migration.
